# Dl-3-n-butylphthalide Reduces Neurovascular Inflammation and Ischemic Brain Injury in Mice

**DOI:** 10.14336/AD.2019.0608

**Published:** 2019-10-01

**Authors:** Chun-Sheng Yang, Ai Guo, Yulin Li, Kaibin Shi, Fu-Dong Shi, Minshu Li

**Affiliations:** ^1^Department of Neurology, Tianjin Neurological Institute, Tianjin Medical University General Hospital, Tianjin 300052, China; ^2^Department of Neurology, Barrow Neurological Institute, St. Joseph’s Hospital and Medical Center, Phoenix, AZ 85013, USA

**Keywords:** ischemic stroke, dl-3-n-butylphthalide, neurovascular unit, neuroinflammation

## Abstract

Dl-3-n-butylphthalide (NBP) is a synthetic compound that has been approved for the treatment of ischemic stroke in China. The mechanisms underlying the treatment efficacy of NBP have been reported in multiple studies and remain controversial. Here, we show that NBP treatment attenuated ischemic brain injury in mice subjected to transient middle cerebral artery occlusion or photothrombosis-induced permanent cerebral ischemia. NBP induced downregulation of intercellular adhesion molecule 1 and protease-activated receptor 1 in cerebrovascular endothelial cells after cerebral ischemia and reperfusion. This effect was associated with the reduced brain infiltration of myeloid cells and improved cerebral blood flow after reperfusion. The beneficial effects of NBP were diminished in mice subjected to the depletion of Gr1^+^ myeloid cells before brain ischemia. Therefore, the restriction of neurovascular inflammation is a key mode of action for NBP in ischemic stroke.

Intravenous thrombolysis with recombinant tissue plasminogen activator (rtPA) and mechanical throm-bectomy are currently two standard treatments for acute ischemic stroke [1, 2]. However, due to restrictive inclusion criteria, the invasiveness of the techniques, and severe adverse events, fewer than 15% of patients benefit from these treatments [3-6]. The development of alternative or adjunctive treatments that can benefit the majority of patients is an urgent need in stroke management.

Brain ischemia swiftly triggers immune responses that involve multiple cellular and molecular participants within the cerebrovascular compartment [7-9]. The interaction among the cerebrovascular endothelium, leukocytes, and platelets exacerbates thrombosis and cerebrovascular dysfunction, leading to infarct expansion and worsened neurological outcome [10-16]. During such process, endothelial cells actively participate in neurovascular inflammation after stroke. As early as hours after brain ischemia, increased oxidative stress activates endothelial cells to a proinflammatory and prothrombotic state, and several proteins, such as protease activated receptor 1 (PAR-1), vascular cell adhesion molecule 1 (VCAM-1), and tissue factor (TF), are upregulated in the ischemic core and surrounding tissue [17-19]. Activated endothelial cells then facilitate the adhesion and accumulation of leukocytes in the cerebrovasculature followed by parenchymal infiltration, leading to exacerbated inflammation within neuro-vascular unit and accelerated infarct expansion.

Dl-3-n-butylphthalide (NBP) is a synthetic compound based on l-3-n-butylphthalide that can be extracted from the seeds of Apium graveolens Linn. Synthesized NBP has been approved for the treatment of ischemic stroke patients with medium and small infarct foci in China [20]. In experimental stroke models, NBP was reported to possess anti-platelet [21] and anti-apoptosis [22] activities, as well as the promotion of neural regeneration [23, 24]. In addition, NBP treatment preserved cerebral blood flow [25, 26], improved BBB integrity [26] and reduced cerebrovascular thrombosis [25]. However, the mechanisms underlying this process remain unknown, and few studies have focused on the inflammatory state of the endothelium and vascular inflammation. In this study, we hypothesized that the benefit of NBP against ischemic brain injury involves the restriction of cerebrovascular inflammation by decoupling the interaction between invading leukocytes and neurovascular unit. We examined the effects of NBP on neurovascular inflammation in two murine models of cerebral ischemia.

## MATERIALS AND METHODS

### Animals

For all experiments, male C57BL/6 mice (7-8 weeks old) were purchased from Beijing Vital River Laboratory Animal Technology Co., Ltd. (Beijing, China). All animal experiments were approved by the Institutional Animal Care and Use Committees of Tianjin Medical University General Hospital. This study was conducted according the National Institutes of Health (NIH; Bethesda, MD, USA) Guide for the Care and Use of Laboratory Animals. All experiments were designed, performed, and reported according to the Animal Research: Reporting of *In Vivo* Experiments guidelines [27, 28]. The mice were maintained in an animal facility under a standardized light-dark cycle with free access to food and water. The animals were randomly assigned to individual groups.

### Mouse models of ischemic brain injury

#### Middle cerebral artery occlusion (MCAO) procedure

A model of transient 60-min intraluminal occlusion of the middle cerebral artery (MCAO) was conducted using the filament method, as previously described [29, 30]. Briefly, the mice were anesthetized by the inhalation of 3.5% isoflurane, and the anesthetic plane was maintained through the inhalation of 1.0-2.0% isoflurane in 70% N_2_O and 30% O_2_ through a nose cone. A standardized silicone rubber-coated nylon monofilament (MSMC21B104 PK50, RWD Life Science, Shenzhen, China) was inserted into the right MCA to occlude circulation for 60 min, and reperfusion was reestablished when the occluding filament was withdrawn gently to the common carotid artery. Cerebral blood flow (CBF) was monitored by a laser Doppler probe (model P10, Moor Instruments, Wilmington, DE, USA) for 5 min both before and after MCAO as well as during the first 10 min of reperfusion. Only MCAO mice with a residual CBF of < 20% of the preischemic levels during the ischemic period and recovery levels of > 80% within 10 min of reperfusion were included in our study. During the surgical procedures, body temperature was maintained using an electric warming blanket.

#### Photothrombotic stroke procedure

Photothrombotic occlusion was performed as previously reported [31]. Briefly, the mice were anesthetized and placed in a stereotactic apparatus (RWD Life Science, Shenzhen, China). The skull was exposed by a midline incision and was cleared of connective tissue and dried. A cold light source (KL1600 LED, SCHOTT AG, Mainz, Germany) filtered with a green filter that provided a 2-mm diameter illumination area was positioned over the top of the skull centered rostrocaudally and 2?mm lateral to bregma. A rubber mask with a small aperture was used to restrict the illuminated area. Next, 150 mg/kg rose bengal dye (Sigma-Aldrich, St. Louis, MO, USA) in saline was administered by i.p. injection. After 5?min, the brain was illuminated through the intact skull for 15?min. Following surgery, the wound was closed using a suture. The animals were continuously monitored until normal function was recovered.

### Drug administration

Synthesized NBP was generously provided by Shijiazhuang Pharmaceutical Group Ouyi Pharma Co., Ltd, (Shijiazhuang, China). NBP was dissolved in vegetable oil and given to MCAO mice immediately after reperfusion at a dose of 60 mg/kg via oral gavage. Mice that received an equal volume of vehicle (oil) were used as controls. To deplete neutrophils and monocytes/ macrophages *in vivo*, the mice were treated with an anti-mouse Gr-1 monoclonal antibody (MAb-RB6-8C5, BioXcell, West Lebanon, NH, USA). The anti-Gr-1 mAb (250 µg per mouse) was administered intraperitoneally one day before MCAO induction and 1 day after surgery [32]. Isotype IgG (rat IgG2b isotype control, BioXcell, West Lebanon, NH, USA) was given to the control group. The RB6-8C5 monoclonal antibody used in this study reacted strongly with mouse Ly6G and to a lesser extent with mouse Ly6C.

### Assessment of infarct volume

#### 2,3,5-Triphenyltetrazolium chloride (TTC) staining

On days 1 and 3 after MCAO and reperfusion, TTC staining was performed to evaluate the infarct volume. Whole brains were harvested rapidly after PBS perfusion. Following incubation for 15 min at -20°C, the frozen whole brains were cut into 1-mm-thick coronal slices using a mouse brain slicer (Zivic Instruments, Pittsburgh, PA, USA). A 2% (w/v) TTC solution (Sigma-Aldrich, St. Louis, MO, USA) was used to stain brain sections for 20 min at 37°C. The infarct area was determined by measuring the regions that lacked TTC staining, which was quantified using Image Pro Plus analysis, as previously described [29].

#### MRI imaging

In some experiments, the infarct volume was assessed using a 7T MRI Scanner (Bruker, Corp., Billerica, MA, USA) equipped with a 72-mm linear transmitter coil and mouse surface receiver coil, as previously described [30, 33]. The mice were anesthetized by inhaling 3.5% isoflurane, and the anesthetic plane was maintained with 1.0-2.0% isoflurane in 70% N_2_O and 30% O_2_. The mice were positioned on a heated circulating water blanket to maintain their body temperatures. T2-weighted images of the brain were acquired with a fat-suppressed rapid acquisition with relaxation enhancement (RARE) sequence to assess the infarct volume (TR = 4000 ms, TE = 60 ms, field of view (FOV) = 19.2 × 19.2 mm, image matrix = 192 × 192, slice thickness = 0.5 mm). The MRI data were analyzed with ImageJ software (National Institutes of Health, Washington, DC, USA).

### Neurological Function Assessment

A battery of neurological tests, which included the modified Neurological Severity Score (mNSS) [34], the corner turning test [35] [30], the foot fault test [36], and the rotarod test [37], was used to assess neurological deficits at the indicated time points. The neurological deficit assessments were performed by investigators blinded to the treatment groups.

#### Modified Neurological Severity Score (mNSS)

The mNSS test included a set of tasks to evaluate many aspects of neurological functions, such as motor function and functional sensory reflexes. The score range for the mNSS was 0-18. The rating scores were interpreted as follows: a score of 13-18 indicated severe injury; 7-12 indicated moderate injury; and 1-6 indicated mild injury. One point was given if the mouse failed to perform a test or lacked a tested reflex. The mNSS assessment was performed by two investigators who were blinded to the treatment groups, as previously described [34].

#### Corner turning test

The corner turning test was performed to evaluate sensorimotor and postural asymmetries. Each mouse was allowed to go to the corner between two boards joined at a 30° angle. The test mouse would turn right or left at the wedge of the corner, and the investigators recorded the direction it turned and repeated the procedure 10 times with at least 30 s between trials. The percentage of ipsilateral turns was calculated.

#### Foot fault test

The foot fault test was performed to assess sensorimotor function after surgery, as previously described [36]. The mice were allowed to walk on a grid with a mesh size of 12 mm for 5 min. A foot fault was recorded when a limb dropped into a hole in the grid. The percentage of foot faults was calculated as follows: foot fault / (foot fault + non foot fault steps) x 100.

#### Rotarod test

The rotarod test was performed to evaluate systemic motor function, especially for coordination and balance. As previously reported [37], the mice were placed on a rotarod apparatus (3 cm in diameter and 30 cm long with a nonslip surface 20 cm above the base). The rod’s rotational speed was accelerated from 0 to 40 rpm. The amount of time each mouse spent on the rod was recorded. Three trials were conducted, and the results were calculated as the average of three trials.

### Hematoxylin & eosin (H&E) staining

H&E staining was performed to measure the occluded vessel index in ischemic hemisphere as previously described [38]. Three days after MCAO, the mice were perfused with cold PBS and fixed with 4% paraformaldehyde. Then, 10-μm-thick coronal sections were prepared with a microtome (Leica Biosystems, Nussloch, Germany). H&E staining was performed by trained histology laboratory personnel. For quantification, three sections of each brain were imaged using a microscope (Olympus, Tokyo, Japan), and the occluded blood vessels within the ischemic hemispheres were counted by two investigators who were blinded to the groups. The occluded vessel index was defined as the ratio of occluded vessels to the total vessels in the ipsilateral hemisphere.

### Cortical cerebral blood flow (CBF) measurements

Cortical CBF was monitored with a laser speckle technique, as previously described [39]. Briefly, images were acquired with a laser speckle contrast imager (PeriCam PSI System, Stockholm, Sweden). Mice treated with the vehicle and NBP were subjected to CBF measurements on day 3 after MCAO. We used the PeriCam PSI HD system (Perimed, Sweden) to calculate an arbitrary index of cerebral blood flow (perfusion units) in the ipsilateral hemisphere.

### Evans blue permeability assay

Blood brain barrier damage was measured by Evans blue permeability assay. In brief, a solution of 2% Evans blue (Sigma-Aldrich, St. Louis, MO, USA) was intravenously injected into mice on day 3 after MCAO. The mice were sacrificed 2 h after injection, and the brains were harvested and weighed. The brain tissues were then homogenized in 5 mL of formamide and incubated in a 60°C water bath for 72 h. After the samples were centrifuged, the optical density of the supernatant was read with a microplate reader (Thermo Scientific, Waltham, MA, USA). The EB concentration was calculated based on a standard curve. The following formula was used: EB content in brain tissue (μg/mg wet brain) = EB concentration (μg/ml) x formamide (ml) / wet weight (mg).

### Flow cytometry

On day 3 after MCAO, mouse brain tissues were harvested and digested with collagenase IV to form a single cell suspension. After the myelin was removed with a 30% Percoll solution, the cells were stained with fluorochrome-conjugated antibodies, as previously described [32]. Briefly, all antibodies were purchased from BioLegend (San Diego, CA, USA) unless otherwise indicated. The following antibodies were used: anti-CD45 (30-F11), anti-CD11b (M1/70), anti-CD3 (145-2C11), anti-CD4 (GK1.4), anti-CD8 (53-6.72), anti-NK1.1 (PK136), anti-CD19 (1D3), anti-F4/80 (6F12), anti-Ly6G (1A8), anti-Ly6C (HK1.4), anti-PAR1(BD Biosciences, San Jose, CA, USA ) , anti-claudin-5 (Abcam, Cambridge, MA, USA) and anti-ZO-1 (Abcam, Cambridge, MA, USA). An Alexa Fluor® 488-labeled donkey anti-rabbit IgG secondary antibody (Invitrogen, Carlsbad, CA, USA) was used for the final detection of the primary antibodies for PAR1, Claudin 5 and Zo-1. Flow cytometry was performed on a FACSAria flow cytometer. The data were analyzed using Flow Jo 7.6.1 software.

### Real-time PCR

On day 3 after MCAO, total mRNA from the brain tissue of the ischemic hemisphere was extracted using Trizol reagent (Invitrogen, Carlsbad, CA, USA). Next, 1 µg of mRNA was reverse transcribed into cDNA using the PrimeScript^TM^ RT reagent kit (TaKaRa, Shiga, Japan). For in vitro experiments, bEnd3 cells were harvested, and then mRNA was extracted, and reverse transcribed into cDNA as described above. SYBR Green PCR Master Mix (Roche, Indianapolis, IN, USA) was used to amplify the gene sequences on the Opticon 2 Real-Time PCR Detection System (BioRad, Hercules, CA, USA). GAPDH served as a reference gene. The primers used in our study are listed in Table 1:

**Table 1:** Primers used in the study.

Gene names	Primer sequence
IL-1β	Forward: 5’-ACGCTTACCATGTGAGCTG-3’ Reverse: 5’-GCCACAGGGATTTTGTCGTT-3’
iNOS	Forward: 5’-GACGAGACGGATAGGCAGAG-3’ Reverse: 5’-CACATGCAAGGAAGGGAACT-3’
IL-6	Forward: 5’-AGCCAGAGTCCTTCAGAGAG-3’ Reverse: 5’-AGGAGAGCATTGGAAATTGGGG-3’
CCL2	Forward: 5’-CTGCTGTTCACAGTTGCCG-3’ Reverse: 5’-GCACAGACCTCTCTCTTGAGC-3’
CCL3	Forward: 5’-AGATTCCACGCCAATTCATC-3’ Reverse: 5’-CCCAGGTCTCTTTGGAGTCA-3’
IFN-γ	Forward: 5’-ATCAGGCCATCAGCAACAA-3’ Reverse: 5’-ACCTGTGGGTTGTTGACCTC-3’
TNFα	Forward: 5’-CGGGCAGGTCTACTTTGGAG-3’ Reverse: 5’-ACCTGGACATTACGACCCT-3’
MMP-9	Forward: 5’-AGACGACATAGACGGCATCC-3’ Reverse: 5’-TGGGACACATAGTGGGAGGT-3’
ZO-1	Forward: 5’-CCACCTCGCACGCATCACAG-3’ Reverse: 5’-TGGTCCTTCACCTCTGAGCACTAC-3’
PAR-1	Forward: 5’-AGCAGACCATCTACATTCCAGCATTG-3’ Reverse: 5’-TGAGTGTTCATCCATAGCAGAAGAGC-3’
ICAM-1	Forward: 5’-ACCCAACTGGAAGCTGTTTG-3’ Reverse: 5’-CACACTCTCCGGAAACGAAT-3’
GAPDH	Forward: 5’-GCCAAGGCTGTGGGCAAGGT-3’ Reverse: 5’-TCTCCAGGCGGCACGCAGA-3’

### Hypoxic glucose deprivation (HGD)

To mimic ischemic conditions, a murine endothelial cell line (bEnd3, kindly provided by Professor Luyuan Li of Nankai University, Tianjin, China) was exposed to HGD, as described previously [40, 41]. In short, bEnd3 cells were seeded in a 12-well culture plate with 3x10^5^ cells per well and cultured at 37°C in a CO_2_ incubator for 24 h. Before HGD, all dishes were washed twice with glucose-free Hanks’ solution, and 1 ml of Hanks’ solution was added to each well. For NBP treatment, 100 μM NBP and an equal volume of vehicle were added to the indicated wells. For HGD, the plates were transferred to a chamber containing 5% CO_2_ and 95% N_2_ for 5 h at 37°C. After HGD, we replaced the Hanks’ solution with cell culture medium (high glucose DMEM +10% FBS) with 100 μM NBP and vehicle and then cultured the cells in a CO_2_ incubator for another 24 h.

### Statistical analysis

The results were analyzed by investigators blinded to the treatment groups. The data are shown as the means ± SEM. Statistical analyses were performed using GraphPad Prism 6.0 software. A two-tailed unpaired Student’s t-test was used to determine the significance of the differences between two groups. One-way ANOVA followed by a Tukey post hoc test were used for 3 or more groups. Two-way ANOVA accompanied by a Bonferroni *post hoc* test was performed for multiple comparisons. *P* values < 0.05 were considered statistically significant.

**Figure 1.**
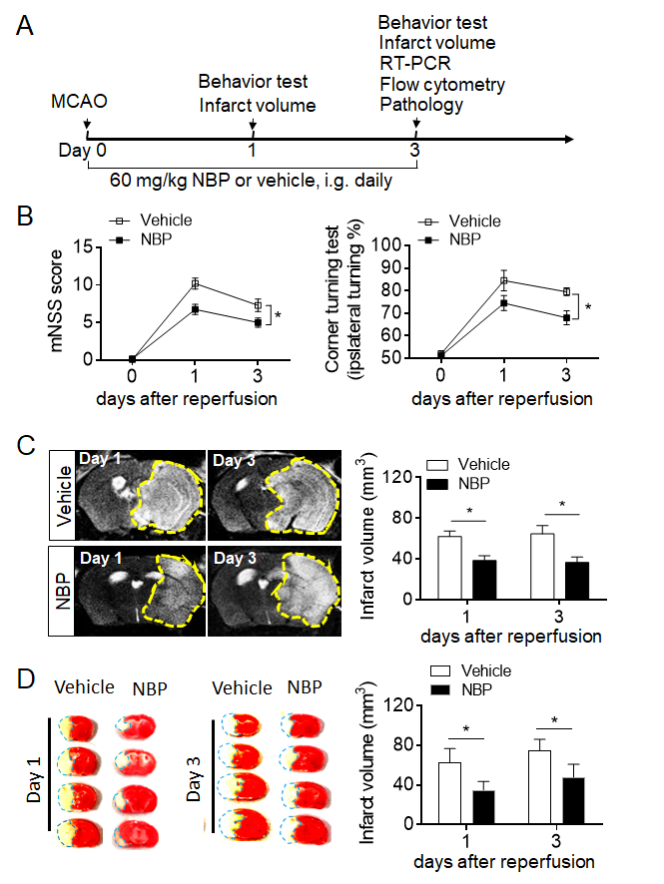
NBP treatment ameliorates neurological deficits and brain infarction after cerebral ischemia and reperfusion. (A) Experimental scheme to assess the impact of NBP on neurological function and infarct volume in mice subjected to 60-min MCAO. NBP (60 mg/kg) or vehicle (oil) were given immediately after reperfusion by oral gavage (i.g.) and repeated daily for three consecutive days. Neurological deficits were evaluated with the modified neurological severity score (mNSS) and corner turning test. Infarct volume was quantified on days 1 and 3 using 7T MRI combined with TTC staining. (B) Summarized results showing the mNSS scores and corner turning test results of MCAO mice receiving NBP or vehicle at the indicated times after reperfusion. The data are expressed as the mean ± SEM. n = 10 per group. *P < 0.05, two-way ANOVA. (C) Infarct volume was quantified with MRI (T2WI) on days 1 and 3 after ischemia. The images show the infarct areas (yellow dashed line). n = 5 mice per group. *P < 0.05, two-tailed unpaired Student’s *t* test. (D) TTC-stained brain slices showing the infarct areas (blue dashed lines) of mice receiving NBP or vehicle at the indicated times after reperfusion. n = 10 mice per group. *P < 0.05, two-tailed unpaired Student’s t test. The data are representative of three independent experiments. Mean ± SEM

## RESULTS

### NBP attenuates acute brain inflammation and ischemic brain injury in mice

**Figure 2.**
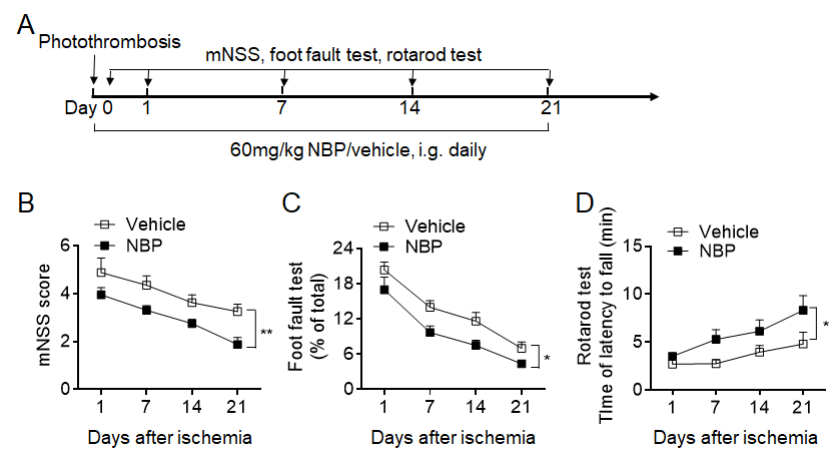
NBP improves long-term outcomes after photothrombosis-induced permanent cerebral ischemia. Cerebral ischemia was induced by photothrombosis in groups of mice receiving NBP or vehicle. (A) Experimental scheme used to assess the impact of NBP on neurological function in mice subjected to photothrombosis. NBP (60 mg/kg) or vehicle was administered daily by i.g. for 21 days. A battery of neurological tests, including mNSS, the foot fault test and the rotarod test, was used to comprehensively evaluate the motor and sensory deficits of the mice 1, 7, 14 and 21 days after surgery. (B-D) Graphs showing the results of mNSS (B), the foot fault test (C), and the rotarod test (D) for the groups of mice receiving NBP or vehicle control until day 21 after photothrombosis. n = 8 per group. *P < 0.05, **P < 0.01, two-way ANOVA. The data are representative of three independent experiments. Mean ± SEM.

To study the effect of NBP on ischemic stroke and investigate its potential mechanism, mice that underwent 60-min middle cerebral artery occlusion (MCAO) were used. The experimental scheme, including the assessment of outcomes and the experimental designs, is illustrated in Figure 1A. Our data show that, compared to vehicle control, NBP significantly reduced neurological deficits and the infarct volume on days 1 and 3 after MCAO (Fig. 1B-D). We then tested NBP in a murine cortical ischemia model induced by photothrombosis, in which the infarct was restricted to the cortical area of the middle cerebral artery territory. NBP attenuated acute neurological deficits and improved motor function recovery up to 3 weeks after ischemia (Fig. 2A-D). Taken together, these results suggest that NBP attenuates acute ischemic brain injury and improves functional recovery after ischemic stroke in mice.

**Figure 3.**
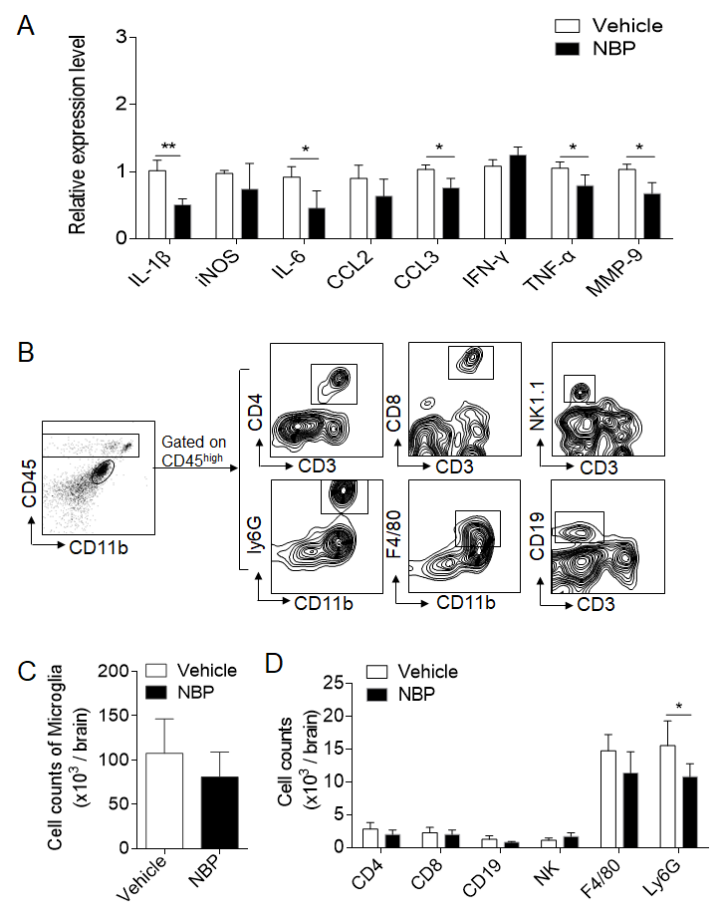
NBP treatment attenuates brain inflammation after cerebral ischemia and reperfusion. MCAO mice were given NBP (60 mg/kg) or vehicle i.g. immediately after 60 min MCAO and reperfusion. Mice received daily treatment until they were sacrificed. (A) RT-PCR detected cytokine levels in the brain tissue homogenates of the ipsilateral hemisphere from the indicated groups of mice. n = 4 per group. *P < 0.05, **P < 0.01, two-tailed unpaired Student’s* t* test. (B) Counts of CNS-infiltrating immune cell subsets and microglia were measured using flow cytometry on day 3 after reperfusion. The gating strategies of immune cell subsets, including macrophages (CD45^high^CD11b^+^F4/80^+^), neutrophils (CD45^high^CD11b^+^Ly6G^+^), microglia (CD11b^+^CD45^inter^), CD4^+^ T cells (CD45^high^CD3^+^CD4^+^), CD8^+^ T cells (CD45^high^CD3^+^CD8^+^), NK cells (CD45^high^CD3^-^NK1.1^+^), and B cells (CD45^high^CD3^-^CD19^+^). (C-D) Summarized results showing the cell counts of the indicated subsets. n = 6 mice per group. *P < 0.05, two-tailed unpaired Student’s *t* test. The data are representative of three independent experiments. Mean ± SEM.

We next determined whether NBP can impact brain inflammation. RT-PCR data showed that the gene expression of pro-inflammatory factors, including IL-6, IL-1β, TNFα, CCL3 and MMP-9, was significantly reduced in the brain tissues of MCAO mice treated with NBP compared to those of control mice (Fig. 3A). We then tested whether NBP treatment can impact immune cell infiltration. Flow cytometric results showed that NBP reduced the brain infiltration of myeloid cells, especially neutrophils (CD45^high^CD11b^+^Ly6G^+^), on day 3 after ischemia (Fig. 3B-D). No significant changes in lymphocytes or resident microglia were observed (Fig. 3B-D). These results indicate that NBP attenuates the inflammatory milieu of the ischemic brain during the acute stage.

### NBP treatment preserves cerebrovascular function after brain ischemia

**Figure 4.**
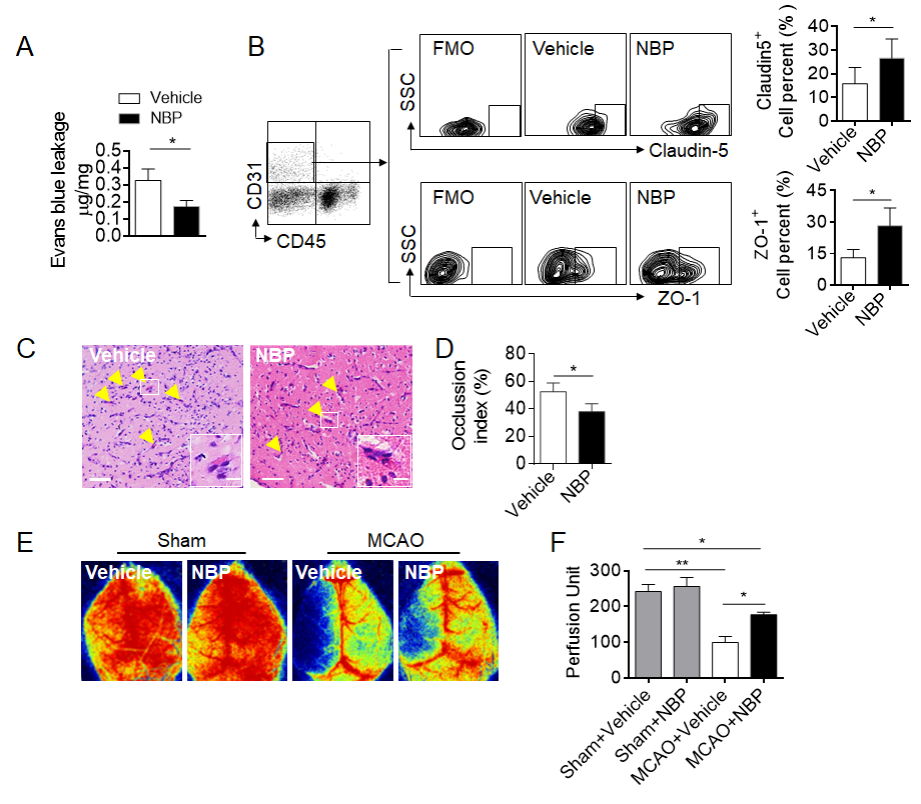
NBP treatment preserves BBB integrity and improves cerebral blood flow after brain ischemia. Mice were subjected to 60-min MCAO. NBP treatment by i.g. (60 mg/kg daily for three consecutive days) was initiated immediately after reperfusion. MCAO mice receiving vehicle were used as controls. The cerebral blood flow (CBF) and blood brain barrier leakage of the MCAO mice were measured using laser speckle Doppler and Evans blue extravasation, respectively, on day 3 after reperfusion. The brains of the mice were then harvested for pathology or flow cytometry analysis. (A) Quantification of Evans blue dye leakage on day 3 after MCAO in the indicated groups. EB content in brain tissue (μg / mg wet brain) = EB concentration (μg/ml) x formamide (ml) / wet weight (mg). n = 3 per group, two-tailed unpaired Student’s* t* test. (B) Flow cytometry analysis of the expression of tight junction proteins (ZO-1 and claudin-5) in endothelial cells from mice receiving NBP or vehicle. FMO, fluorescence minus one control. n = 6 per group. *P < 0.05, two-tailed unpaired Student’s *t* test. (C-D) H&E images showing occluded vessels in the ischemic brains of MCAO mice from the indicated groups. The bar graph shows the occlusion index of occluded vessels in the ischemic brains of MCAO mice. The occlusion index was calculated as the percentage of occluded vessels among the total observed vessels in the ischemic brain. Scale bars: 50 µm, 10 µm in the insets of C. n = 4 per group. *P < 0.05, two-tailed unpaired Student’s *t* test. (E) Images of CBF in sham-operated and MCAO mice that received NBP or vehicle treatment. (F) Quantification of blood perfusion in the ipsilateral hemisphere. n = 6 per group. *P< 0.05, **P < 0.01, one-way ANOVA. The data are representative of two independent experiments. Mean ± SEM.

The dysfunction of the microvasculature is characterized by BBB disruption and inflammatory microthrombus formation. These are key pathological processes that cause secondary brain injury after stroke, leading to penumbra shrinkage and infarct expansion [10, 42]. Flow cytometric data showed that, compared to vehicle control, NBP treatment preserved the expression of the tight junction proteins ZO-1 and claudin-5 in brain endothelial cells (Fig. 4B), resulting in the maintenance of BBB integrity, as indicated by reduced Evans blue extravasation on day 3 after MCAO (Fig. 4A). In addition, histological staining showed that occluded microvessels in the ipsilateral hemisphere were significantly reduced in the MCAO mice treated with NBP compared to those treated with vehicle, and this effect was accompanied by improved cerebral blood flow (Fig. 4C-F). These data suggest that NBP preserves the integrity and patency of brain vasculature after ischemia, which leads to improved cerebral blood flow recovery after reperfusion.

**Figure 5.**
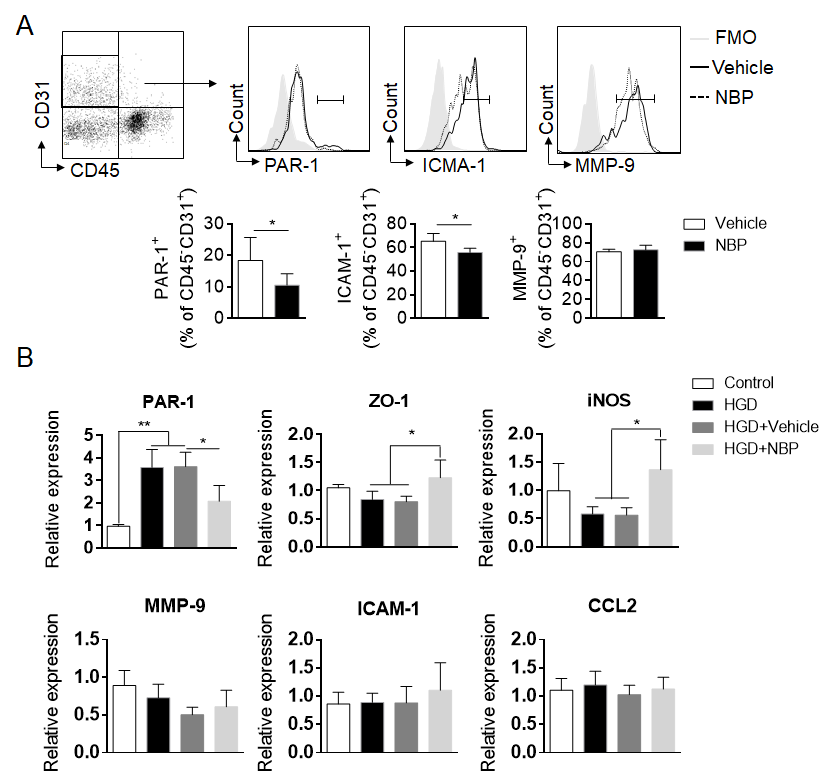
NBP modulates the activity of cerebrovascular endothelial cells during brain ischemia. (A) Flow cytometry analysis of the expression of thrombin receptors (PAR-1), ICAM-1 and MMP-9 in brain endothelial cells from MCAO mice treated with NBP or vehicle control on day 3. n = 6 per group. *P < 0.05, two-tailed unpaired Student’s *t* test. (B) The effect of NBP treatment on the expression of the indicated mRNA in bEnd3 cells (a mouse brain endothelial cell line) after exposure to HGD *in vitro*. bEnd3 cells were exposed to HGD for 5 h in the presence of 100 µM NBP or vehicle (20% cyclodextrin in saline). After 24 h in culture, the cells were harvested for RT-PCR analysis. n = 4 per group. *P < 0.05, **P < 0.01, one-way ANOVA. The data are representative of three independent experiments. Mean ± SEM.

The activation of brain microvascular endothelial cells following stroke is essential to the initiation of vascular inflammation and the subsequent brain infiltration of peripheral immune cells. Almost immediately after ischemic insult, oxidative triggering factors activate pro-inflammatory, pro-coagulation, and pro-permeability features through vascular endothelial cells by upregulating a series of protein products, including ICAM-1, CXCL2, MMP-9, and PAR-1. This process facilitates leukocyte adhesion, microthrombus formation and BBB disruption [19, 43]. Thus, we evaluated whether NBP treatment can modulate the activation status of the endothelium. NBP treatment significantly inhibited the upregulation of PAR-1 and ICAM-1 in brain endothelial cells after ischemia (Fig. 5A). We then tested whether NBP can directly target endothelial cells in an *in vitro* endothelial model of hypoxic glucose deprivation (HGD). HGD significantly upregulated PAR-1 expression in endothelial cells and reduced ZO-1 expression. NBP treatment significantly inhibited PAR-1 upregulation and preserved ZO-1 expression after HGD in endothelial cells (Fig. 5B). These results together suggest that NBP can inhibit intravascular inflammation through the modulation of endothelial cell activation.

### Gr1^+^ cell depletion diminishes the beneficial effects of NBP treatment after brain ischemia

**Figure 6.**
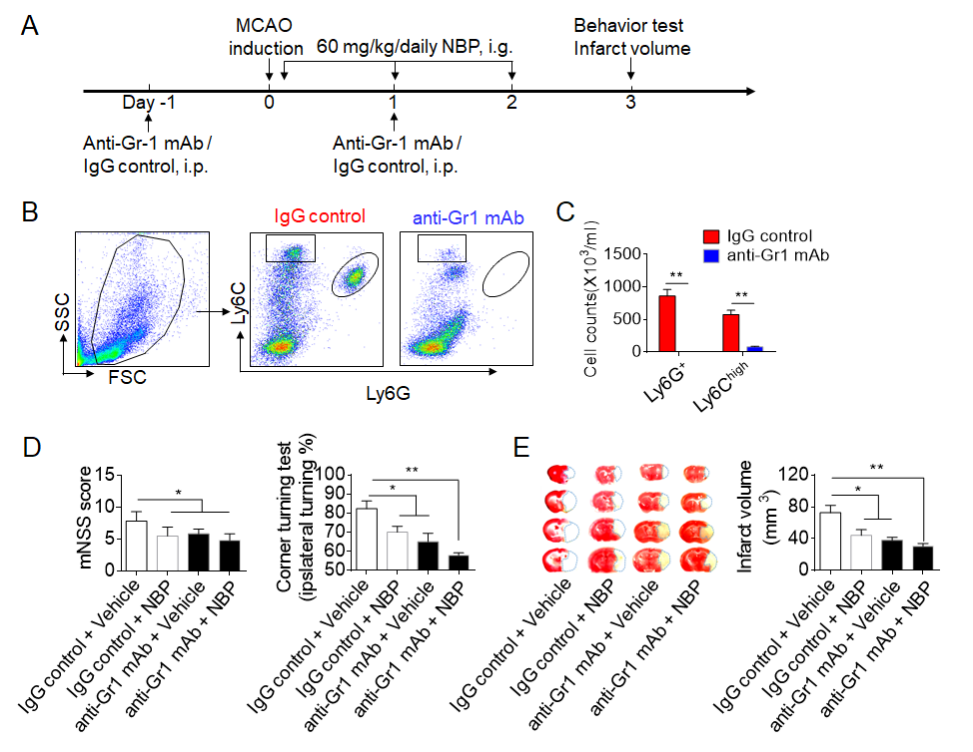
The benefit of NBP on ischemic stroke involves its effects on Gr1^+^ cells. (A) Schematic diagram illustrating the drug administration and experimental design. C57BL/6 mice received anti-Gr-1 mAb or IgG control 1 day prior to and 1 day after the MCAO procedure. NBP or an equal volume of vehicle were administered by i.g. (60 mg/kg daily for three consecutive days) immediately after reperfusion. On day 3 after MCAO, neurological deficits and the infarct volume were assessed. (B-C) Flow cytometric determination of absolute numbers of circulating Ly6G^+^ and Ly6C^high^ cells from the mice that received anti-Gr-1 mAb or IgG control on day 3 after MCAO. n = 4 mice per group, **P < 0.01, two-tailed unpaired Student’s *t* test. (D) Summarized results of the mNSS and the corner turning test of MCAO mice treated with NBP or vehicle control with or without Gr1^+^ cell depletion. n = 6 mice per group. (E) Representative coronal brain sections stained with TTC and the quantification of the infarct size of mice receiving NBP or vehicle with or without Gr1^+^ cell depletion on day 3 after MCAO. n = 6 mice per group. *P < 0.05, **P < 0.01, one-way ANOVA. The data are representative of three independent experiments. Mean ± SEM.

As NBP treatment reduced the brain infiltration of myeloid cells such as neutrophils after ischemia, we then examined the potential involvement of these myeloid cells in the benefit of NBP. An anti-Gr1^+^ mAb which was used to deplete neutrophils and monocytes in mice prior to 60 min MCAO (Fig. 6A). Approximately 90% of Gr1^+^ cells (Ly6G^+^/ Ly6C^high^) were depleted in the anti-Gr1^+^ mAb-treated mice, of which neutrophil (Ly6G^+^) counts decrease 100%, monocytes (Ly6C^high^) counts decrease about 86% (Fig. 6B-C). The depletion of Gr1^+^ cells diminished the benefits of NBP after MCAO and reperfusion (Fig. 6D-E), suggesting that the beneficial effects of NBP treatment involve Gr1^+^ cells.

## DISCUSSION

In this study, we showed that NBP attenuates ischemic brain injury and improves neurological outcomes in two experimental stroke models with different lesion sizes and locations. We demonstrated that NBP is capable of directly targeting cerebral vascular endothelial cells to inhibit the activation of vascular inflammation, thereby reducing the migration of myeloid cells, preserving BBB integrity and microvascular patency. This process leads to improved cerebral blood flow recovery and reduced brain injury. In addition, the benefit of NBP involves Gr1^+^ myeloid cells.

Increasing evidence has demonstrated that inflammation exacerbates secondary brain injury and worsens stroke outcome [44, 45]. Inflammation within the cerebrovascular compartment involves multiple pathological events that include the activation of neruoglia and endothelial cells, the recruitment of leukocytes, and the release of inflammatory mediators such as reactive oxygen species (ROS), matrix metalloproteinase (MMPs), cytokines and chemokines. As endothelial activation is a pivotal player during the initiation of neurovascular inflammation, BBB disruption, and microvascular dysfunction [46, 47]. The present study provides new evidence that the benefit of NBP treatment in ischemic stroke involves a vascular component.

Endothelial cells play an essential role in the initiation of vascular inflammation following stroke. After ischemic stroke, brain vascular endothelial cells are rapidly converted to prothrombotic and proinflammatory states, and these cells potentiate microvascular damage and parenchymal inflammation [17-19]. In this study, we found that NBP can directly regulate the endothelial cell activation status and preserve tight junction after ischemic stroke. This finding is consistent with previous studies showing that NBP preserves BBB integrity in mice subjected to carbon monoxide (CO) poisoning [26]. Additionally, NBP was shown to protect brain microvascular endothelial cells against oxygen glucose deprivation *in vitro* [48]. In line with previous studies, our findings highlight endothelial cells as a potential cellular target of NBP.

Interactions between leukocytes and endothelial cells actively participate in neurovascular inflammation and ischemic brain injury [49-51]. Neutrophils are among the first circulating immune cell populations that infiltrate the ischemic brain and contribute to BBB disruption and neural injury by releasing ROS, MMPs, cytokines (IL-1β, IL-8, IL-6, TNF-α), and chemokines (CCL2, CCL3, CCL5) [8, 52]. In addition, neutrophils may also promote thrombus formation through interactions with platelets, the proteolytic cleavage of clotting factors (TFPI and coagulation factor X), and the release of prothrombotic factors (neutrophil extracellular traps (NETs)) [52, 53]. We found that NBP preferentially reduces the infiltration of myeloid cells including neutrophils after brain ischemia. Of interest, the beneficial effects of NBP treatment are diminished in mice receiving antibody mediated depletion of Gr-1^+^ cells including neutrophils, suggesting that the restricted neurovascular inflammation after NBP treatment involves its effects on Gr-1^+^ cells. Together with these results, our finding of improved cerebral blood flow after NBP treatment further support our hypothesis that NBP improves cerebral circulation via decoupling the invading immune cells and cerebrovascular endothelial cells.

In conclusion, we present new evidence that NBP reduces ischemic brain injury via restriction of neurovascular inflammation.
